# Risk factors affecting intimate partner violence occurrence in South Korea: Findings from the 2016 Domestic Violence Survey

**DOI:** 10.1371/journal.pone.0247916

**Published:** 2021-03-04

**Authors:** Young-Ran Han, Hye Young Choi

**Affiliations:** 1 Department of Nursing, Dongguk University, Gyeongju-si, Gyeongsangbuk-do, Republic of Korea; 2 Department of Nursing, Kangwon National University, Samcheok-si, Gangwon-do, Republic of Korea; Monash University, AUSTRALIA

## Abstract

This study aimed to identify factors affecting the occurrence of intimate partner violence (IPV) in Korean adults aged 19 years and older. Specifically, we identified the factors in women’s victimization in and men’s perpetration of IPV. This study adopted a cross-sectional and correlational design in conducting secondary data analysis of the 2016 Domestic Violence Survey in Korea. Men (N = 1,272) and women (N = 2,689) with partner were included in the analysis. The ecological model was used as a conceptual framework. Multiple logistic regression analyses were conducted to identify factors affecting women’s victimization in and men’s perpetration of IPV. The results showed that the prevalence of IPV against female partner was 12.1%. IPV occurrence was higher among women (Odds ratios (OR) = 2.92, Confidence intervals (CI): 1.84–4.63) and men (OR = 2.64, CI: 1.62–4.32) who experienced witnessing inter-parental violence in childhood, and among women (OR = 2.25, CI: 1.40–3.61) and men (OR = 2.68, CI: 1.59–4.52) with a tolerant attitude toward IPV. The occurrence was higher among women who experienced childhood maltreatment by the parents (OR = 1.70, CI: 1.03–2.82) and women whose income was 2 million Korean Won(KRW) to 3 million KRW compared with women whose income was above 4 million KRW (OR = 1.97, CI: 1.10–3.55). The occurrence was lower among women with office jobs compared with other jobs (OR = 0.47, CI: 0.26–0.84). Based on the results of this study, early intervention in abusive families to reduce the negative impact of abuse experiences and witnessing inter-parental violence in childhood, and education and publicity for changing attitudes toward IPV are necessary at the individual and societal levels. The formation of policies for the stable workplace and income of women are required.

## Introduction

Intimate partner violence (IPV) is the most common type of violence against women (VAW), affecting about 30% of women worldwide [1]. IPV refers to “any behaviour by a man or a woman, or a boy or a girl, within an intimate relationship, that causes physical, sexual, or psychological harm to the other person in the relationship” [1]. IPV represents a serious violation of women’s human rights and an urgent public health priority; it is an important cause of injury, and is a risk factor for many physical and psychological health problems [1,2]. IPV negatively affects the behaviors and emotions of children and often causes severe problems in the family and society through the intergenerational transmission of violence [1,3–5].

The distribution of partner violence shows exceptionally large differences, with the IPV prevalence experienced in the past 12 months in many high-income countries being under 4% and that in some low-income settings being at least 40% [6]. Little is known about the factors that explain the difference in the prevalence of partner violence across countries [6]. Cultural views toward VAW have become a major concern worldwide, yet information remains lacking [7].

Korean society has shown lenient attitudes toward partner violence owing to its patriarchal, male dominated and family-centered culture [8]. However, with the change in the role of women owing to rapid modernization and Westernization, the public attitude toward partner violence is changing [8–10]. In Korea, the Gross National Income per capita has increased to 30,600 USD in 2018 [11]. Meanwhile, the nationwide prevalence of physical, psychological, sexual, or economic violence against the female partner from a male partner in the last year has remained high, at 33.1% in 2007, 39.1% in 2010, and 29.8% in 2013 [12,13]. According to the Korean Women’s Hotline, at least 887 women were victims of homicide by an intimate male partners in 2009–2018, and 727 women survived a murder attempt against them [14]. As there may be more unreported cases, it is likely that the number of victims may exceed official reports.

It is estimated that over 75% of VAW is perpetrated by the male intimate partner [15]. Across a sample drawn from eight low- and middle-income countries, 31% of men report having perpetrated physical violence against a partner in their lifetime [16]. Although there are differences among the countries in the percentage of men who perpetrated physical and/or sexual violence in nine Asian and Pacific countries, the percentage ranges from 25.4% to 80%. When including psychological and/or economic violence, the range rises from 39.3% to 87.3% [17]. Men’s perpetration of VAW results from a complex, interconnected ecology of psychological, economic, and sociological factors [18].

There are many risk factors associated with IPV, such as age, economic status, educational level, marital status, exposure to prior abuse, acceptance of violence and traditional gender roles, and community sanctions [1,5,19,20]. Among other things, gender inequality in the distribution of power and resources and discrimination against women are the main root causes of IPV [1,2,20]. Nevertheless, there are multiple risk factors associated with both perpetration and victimization at multiple levels, such as personal history, personal background, social norms. Risk factors related to the occurrence of IPV differ by country and by the victim and perpetrator of abuse, and the response to them may differ as well [5,16,17,19,20]. In Korea, research on the risk factors related to the occurrence of IPV is lacking and has focused on female victims. Therefore, concurrent research on male perpetrators is necessary and ecological approach that considers various factors related to the occurrence of IPV at the individual, family and social level is useful.

This study aimed to identify the factors that affect the occurrence of IPV from the aspects of both female victim and male perpetrator, based on the socio-ecological theory that involves considering factors contributing to the problem at various levels.

Until recently, VAW was largely invisible within national and international statistics and surveillance systems [20]. Research on IPV was mostly conducted by individual research teams in Korea. The Domestic Violence Survey (DVS) is a triennial study conducted since 2007 by the Ministry of Gender Equality & Family (MGEF) according to the Act on the Prevention of Domestic Violence and Protection, etc. of Victims [13]. Analysis of national survey datasets can be carried out to describe phenomena, generate knowledge for nursing practice, or shed light on the present, the past, or trends over time [21]. Therefore, this study conducted a secondary data analysis (SDA) using the 2016 DVS. The results are expected to provide data that are helpful for policies and interventions for preventing and responding to IPV.

The aim of this study was to identify the factors associated with IPV occurrence in Korean adults. The specific aims were as follows: (1) to identify the prevalence of IPV, (2) analyze differences in women’s victimization in IPV and men’s perpetration of IPV according to multilevel factors, and (3) identify the multilevel factors affecting women’s victimization in IPV and men’s perpetration of IPV.

### Conceptual framework

**Fig 1:**
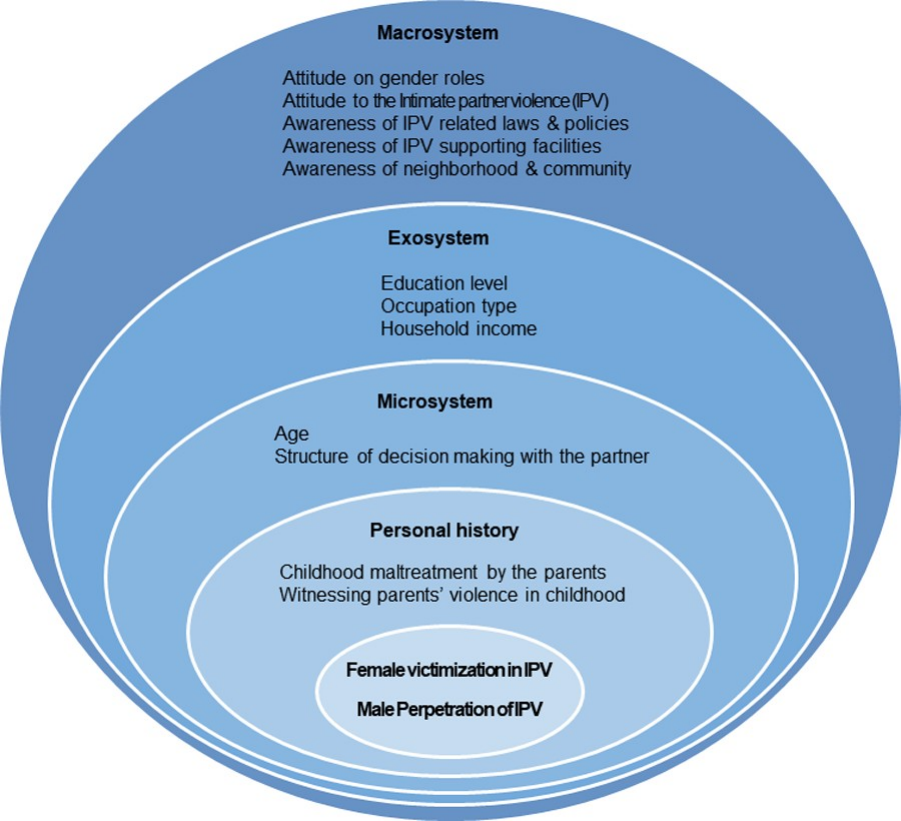
Conceptual framework for this study.

An ecological model was used as the conceptual framework for this study. The ecological model supports a comprehensive public health approach that not only addresses an individual’s risk of becoming a victim or perpetrator of violence but also the norms, beliefs, and social and economic factors that create the conditions for IPV to occur [5,18,22]. The occurrence of IPV is determined by a complex interplay among various factors at each of the ecological layers. This study included witnessing violence between parents or experiencing child abuse by a parent in one’s personal history, age, and structure of decision-making with the partner in the microsystem; education level, occupation type, and household income in the exosystem; and attitude toward gender roles and IPV, awareness of neighborhood and community, awareness of IPV-related laws and policies, and awareness of support facilities in the macrosystem. The conceptual framework for this study is shown in Fig 1.

## Methods

### Design and sample

Our study used a cross-sectional and correlational in design with SDA using the 2016 DVS.

### Description of primary data: The 2016 DVS

The 2016 DVS data was used in this study because it is the only large-scale national survey of IPV in Korea. What is referred as IPV in this study was identified using domestic violence data. The DVS has been conducted to examine the DV experience of general adults. Probability proportion sampling and systematic sampling were applied, with one person selected for each household. After the training of surveyors and field preparation, the survey was conducted by visiting each household and distributing a self-reported questionnaire. From September 22 to December 8, 2016, a total 6,000 adults aged more than 19 years (4,000 women and 2,000 men) were surveyed (95% confidence level, sampling error ± 1.3%p) [23].

### Samples of SDA

**Fig 2:**
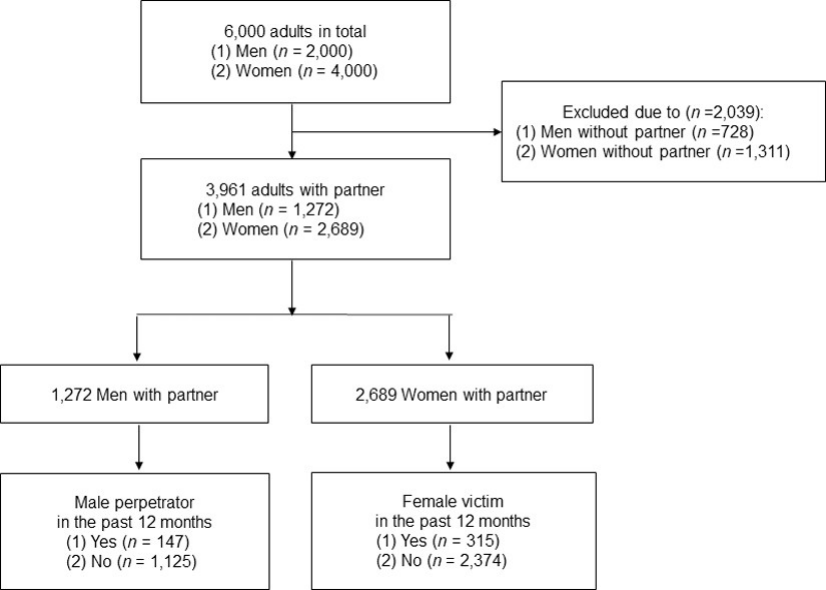
Flow diagram of study sample.

This study analyzed the data of 3,961 participants, composed of 1,272 men and 2,689 women with a partner. The eligibility criterion was that they reported being married or in a common-law marriage. The exclusion criterion was being without a partner (Fig 2).

### Variables

#### Dependent variables

Female victimization in IPV and male perpetration of IPV were set as the dependent variables. IPV was categorized into four types: psychological, physical, economic, and sexual. The Revised Conflict Tactics Scale [24] was reviewed, revised, and complemented to create a list of 15 behaviors; female victim was considered to have occurred if the woman had been subjected to any one of these 15 behaviors in the past 12 months. Likewise, male perpetrator was categorized as having perpetrated violence if the man responded that he engaged in at least one of the 15 items in the past 12 months.

#### Independent variables

*Personal history*. For witnessing inter-parental violence in childhood, if the participant witnessed one or more of the three items when they were 18 years old or younger, the response was classified as “yes.” Items assessed whether the participant has witnessed the following behaviors between their parents: used harsh words, such as swearing or slighting words; struck with a hand or foot; hit or injured with a belt, club, or others. For childhood maltreatment by parents, the participant’s response was classified as “yes” if they experienced one or more of six items related to physical and psychological abuse as well as child negligence by parents when they were aged 18 years or younger. Six items assessed whether the participant heard harsh words, such as swearing or slighting words; was hit on the hand or legs with a cane; was hit with a hand or foot; was hit or injured by a belt, club, or others; was not given food or taken to hospital when sick; and was alone when they should have been with an adult.

*Microsystem*. The structure of decision-making between partners was asked for the person who made the main decision for four items (living expenses, child rearing and education, house purchasing and moving, and investment and property management) on a five-point Likert scale: “1. Completely decided by me,” “2. Mostly decided by me,” “3. Decided together through a discussion,” “4. Mostly decided by my spouse,” and “5. Completely decided by my spouse.” The mean of the four items was determined. A mean greater than 3 points signified that the decision-making power of the spouse was greater.

*Exosystem*. Education level, household income, and occupation type were investigated. Occupation type was classified into “professional and manager,” “office job,” and “other.” Other jobs included positions in services, sales, agriculture, forestry, and fisheries, as well as device and mechanical operation and assembly workers, simple labor workers, and soldiers.

*Macrosystem*. Attitude toward gender roles was measured using a total of seven items on a four-point Likert scale, with higher average scores representing more patriarchal attitude toward gender roles. The seven items included four items on whether the role of the man was to be a leader in society, promote important work, lead sexual relationships, and hold decision-making power over the household finances, and three items on whether wives should mostly do housework, obey the husband’s decision on whether they should have a job, and prioritize the opinion of the husband for important decisions regarding their children. Attitude toward IPV was measured using a total of 10 items on a four-point Likert scale, and higher mean scores indicated a more tolerant attitude toward IPV. The 10 items were composed of three items on whether one can be violent to one’s family if one loses control in anger, if one is under too much stress, or if one drinks too much; two items on whether an abuser can be forgiven if they genuinely repent after the violence or if they were maltreated in childhood; and five items on whether intimate violence is a within-family problem, whether women should endure the violence to protect their family, whether women can leave the relationship if they really want, whether they find it difficult to understand why someone would not leave an abusive spouse, and whether it is correct for the violent spouse to leave home if they engaged in violent behavior. Awareness of IPV-related laws and policies and of support facilities were determined using of six and four items, respectively, and scores were assigned based on the participant’s recognition of each item: 1 for recognition and 0 for non-recognition. Higher total scores represented higher recognition. Awareness of the neighborhood and community was measured using a total of eight items on a four-point Likert scale, and higher average scores represented a higher sense of community.

### Data analyses

All data analyses were conducted with IBM SPSS 24.0. Weighted data were used in the analysis of the report of the 2016 DVS [23]. The weighted data were calculated through three processes, namely, design weight (strata and cluster), adjustment to non-response, and adjustment of using population information.

Descriptive statistics based on frequencies, weighted percentages, and means were generated to describe socio-demographic variables, types of IPV, and other IPV related data. Next, chi-squared tests and *t*-test based on weighted percentages and means were used to analyze the differences in women’s victimization in IPV and men’s perpetration of IPV according to the multi-level factors based on the ecologic model. Subsequently, multiple logistic regression analyses were conducted to identify the factors affecting women’s victimization and men’s perpetration. The results are presented as *p*-values, and 95% confidence intervals (CI).

### Ethics statement

Considering the nature of the survey, the raw data were collected based on WHO guidelines for ethics and safety related to the research and survey of VAW [23]. The investigators of the present study requested raw data from the MEGF based on the Enforcement Rules of the Act on Provision and Active Use of Public Data [25]. To perform the SDA, institutional review board exemption approval was obtained from the Institutional Review Board of the Kangwon National University (approval number: KWNUIRB-2020-01-011).

## Results

### Prevalence of female victimization and male perpetration of IPV

The prevalence of female victimization in IPV in the past 12 months as reported by women was 12.1%. By type, psychological violence comprised 10.5%, physical violence, 3.3%, sexual violence, 2.3%, and economic violence, 2.4%. The prevalence of male perpetration in the past 12 months as reported by men was 11.6%. By type, psychological violence comprised 10.5%, physical violence, 2.1%, sexual violence, 1.8%, and economic violence, 1.5% (Table 1).

**Table 1:** Prevalence of female victimization and male perpetration of IPV(*N* = 3,961).

Variables		Female victims (n = 315)	Male perpetrators (n = 147)
		n (%^b^)	n (%^c^)
IPV Type^a^	Psychological violence	271 (10.5)	131 (10.5)
	Physical violence	87 (3.3)	27 (2.1)
	Sexual violence	64 (2.3)	22 (1.8)
	Economic violence	56 (2.4)	19 (1.5)
	Total	315 (12.1)	147 (11.6)

^a^Multiple responses.

^b^% of female victims among women participants.

^c^% of male perpetrators among men participants.

### Differences in IPV occurrence by multi-level factor

#### Differences in female victimization in IPV by multi-level factor

Participants who witnessed their parents’ violence in childhood (61.8%) and those who experienced childhood maltreatment by their parents (78.8%) had a higher occurrence rate of IPV victimization (*p* < .001, *p* < .001, respectively), compared with participants who did not have these experiences (30.5%, 50.9%, respectively). The IPV victimization rates of participants who graduated middle school (12.4%) and high school (55.3%) were higher (*p* = .034) compared with those without IPV victimization (9.9%, 49.4%). Compared with the comparison group (14.2%), the IPV victimization rate of participants whose income was between 2 and 3 million KRW (22.6%) was higher (*p* = .002). Moreover, compared with the comparison group (71.1%), the IPV victimization rate of participants whose job was classified as “other” (83.0%) was higher (*p* = .005). The mean score for attitude on gender roles among women who were victims of IPV (2.24 ± 0.03) was higher compared with women who were not victims (2.13 ± 0.01), showing that the attitude toward traditional patriarchal gender roles was stronger among victimized women (*p* = .001). The mean score for attitude to IPV among women who were victims of IPV (1.97 ± 0.02) was relatively higher compared with women who were not victims (1.87 ± 0.01), showing that the former have greater tolerance toward and have a lower level of awareness of IPV (*p* < .001) (Table 2).

**Table 2:** Differences in female victimization in IPV by multi-level factor (N = 2,689).

Variables			Total	Female Victimization		*χ*^2^ or t	*p*
				Yes (n = 315)	No (n = 2,374)		
Personal history	Witnessing parents’ violence in childhood	Yes	882 (34.3)	194 (61.8)	688 (30.5)	125.03	< .001
		No	1,807 (65.7)	121 (38.2)	1,686 (69.5)		
	Childhood maltreatment by parents	Yes	1,409 (54.3)	246 (78.8)	1,163 (50.9)	89.83	< .001
		No	1,279 (45.7)	69 (21.2)	1,210 (49.1)		
Micro- system	Structure of decision making with partner	Mean ± SD	2.79 ± 0.02	2.80 ± 0.03	2.77 ± 0.01	1.11	.268
	Age (years)	19–39	793 (23.5)	82 (20.9)	711 (23.9)	2.70	.521
		40–49	720 (27.2)	92 (28.4)	628 (27.1)		
		50–59	691 (26.2)	86 (29.0)	605 (25.9)		
		≥60	485 (23.0)	55 (21.7)	430 (23.2)		
Exo- system	Education level	≤Elementary	204 (8.8)	20 (7.6)	184 (9.0)	10.67	.034
		Middle school	250 (10.2)	40 (12.4)	210 (9.9)		
		High school	1,287 (49.4)	164 (55.3)	1,287 (49.4)		
		≥College	946 (31.6)	91 (24.7)	855 (32.5)		
	Household income (1,000 KRW)	<2,000	309 (12.3)	37 (12.3)	272 (12.3)	17.73	.002
		2,000–2,999	454 (15.2)	75 (22.6)	379 (14.2)		
		3,000–3,999	655 (23.3)	61 (18.1)	594 (24.0)		
		≥4,000	1,268 (49.2)	142 (47.0)	1,126 (49.5)		
	Occupation type	Professional or Manager	80 (5.5)	8 (3.7)	72 (5.8)	5.26	.005
		Office job	340 (21.9)	24 (13.3)	316 (23.1)		
		Others	981 (72.5)	131 (83.0)	850 (71.1)		
Macro- system	Attitude to gender roles	Mean ± SD	2.19 ± 0.02	2.24 ± 0.03	2.13 ± 0.01	3.21	.001
	Awareness of neighborhood and community	Mean ± SD	2.59 ± 0.16	2.60 ± 0.03	2.59 ± 0.01	0.27	.788
	Attitude to the -IPV	Mean ± SD	1.92 ± 0.01	1.97 ± 0.02	1.87 ± 0.01	3.91	< .001
	Awareness of -IPV-related laws and policies	Mean ± SD	3.93 ± 0.06	3.81 ± 0.11	4.05 ± 0.04	-1.94	.053
	Awareness of support facilities	Mean ± SD	2.30 ± 0.04	2.27 ± 0.07	2.33 ± 0.03	-0.88	.377

IPV: Intimate partner violence.

#### Differences in male perpetration of IPV by multi-level factor

Men who witnessed parental violence in childhood (59.5%) and men who experienced childhood maltreatment by their parents (72.4%) had a higher rate of IPV perpetration (*p* < .001, *p =* .002, respectively), compared with men who did not have these experiences (34.0%, 58.1%, respectively). The mean score for attitude toward IPV was higher among men who had experiences with perpetrating violence against an intimate partner (2.05 ± 0.04) compared with men who did not (1.84 ± 0.01), showing that the former had a more tolerant attitude toward and lower awareness of IPV (*p* < .001) (Table 3).

**Table 3:** Differences in male perpetration of IPV by multi-level factor (N = 1,272).

Variables			Total	Male Perpetration		*χ*^2^ or t	*p*
				Yes (n = 147)	No (n = 1,125)		
Personal history	Witnessing parents’ violence in childhood	Yes	451 (37.0)	88 (59.5)	363 (34.0)	36.39	< .001
		No	821 (63.0)	59 (40.5)	762 (66.0)		
	Childhood maltreatment by parents	Yes	741 (59.7)	109 (72.4)	632 (58.1)	11.07	.002
		No	531 (40.3)	38 (27.6)	493 (41.9)		
Micro- system	Structure of decision making with partner	Mean ± SD	3.07 ± 0.03	3.05 ± 0.05	3.09 ± 0.02	-0.95	.345
	Age (years)	19–39	261 (16.8)	24 (14.7)	261 (16.8)	0.61	.913
		40–49	319 (25.8)	40 (27.2)	319 (25.8)		
		50–59	351 (28.6)	45 (29.2)	351 (28.6)		
		≥60	341 (28.7)	38 (28.9)	341 (28.7)		
Exo- system	Education level	≤Elementary	72 (5.5)	9 (5.4)	63 (5.5)	4.42	.302
		Middle school	108 (9.3)	11 (8.9)	97 (9.3)		
		High school	556 (45.1)	76 (52.8)	480 (44.1)		
		≥College	536 (40.2)	51 (32.9)	485 (41.2)		
	Household income (1,000 KRW)	<2,000	186 (14.2)	22 (14.6)	164 (14.2)	1.24	.781
		2,000–2,999	218 (15.6)	23 (14.1)	195 (15.8)		
		3,000–3,999	328 (24.7)	41 (28.2)	287 (24.3)		
		≥4,000	539 (45.5)	61 (43.2)	478 (45.8)		
	Occupation type	Professional or Manager	116 (10.8)	15 (11.9)	101 (10.7)	0.27	.889
		Office job	324 (27.0)	38 (27.8)	286 (26.9)		
		Others	697 (62.2)	78 (60.3)	619 (62.4)		
Macro- system	Attitude to gender roles	Mean ± SD	2.40 ± 0.02	2.43 ± 0.04	2.37 ± 0.02	1.45	.147
	Awareness of neighborhood and community	Mean ± SD	2.58 ± 0.02	2.62 ± 0.04	2.54 ± 0.02	1.62	.105
	Attitude to IPV	Mean ± SD	1.95 ± 0.02	2.05 ± 0.04	1.84 ± 0.01	4.72	〈.001
	Awareness of IPV -related laws and policies	Mean ± SD	4.03 ± 0.08	3.93 ± 0.15	4.13 ± 0.06	-1.28	.201
	Awareness of support facilities	Mean ± SD	2.16 ± 0.06	2.16 ± 0.10	2.16 ± 0.04	0.00	1.000

IPV: Intimate partner violence.

### Factors associated with female victimization and male perpetration of IPV

The results of the multiple regression analysis on the women participants showed that 16.5% of the variance was explained by the four variables of witnessing parents’ violence in childhood, childhood maltreatment by parents, household income, and attitude to IPV. The IPV victimization rates among women who witnessed parental violence in childhood and childhood maltreatment by their parents were 2.92 times (OR = 2.92, CI: 1.84–4.65) and 1.7 times (OR = 2.92, CI: 1.84–4.65) higher than the rates among women who did not have these experiences. Compared with women whose income was above 4 million KRW, women whose income was between 2 and 3 million KRW had an IPV victimization rate that was 1.97 times (OR = 1.97, CI: 1.10–3.55) higher. In terms of jobs, the IPV victimization rate among women who had office jobs was 0.47 times (OR = 0.47, CI: 0.26–0.84) lower compared with women with “other” jobs. In terms of the attitudes toward IPV, each point (1–4 points) increase on the mean attitude toward IPV was found to be associated with a 2.25-fold increase in IPV victimization rate (OR = 2.25, CI: 1.40–3.61) (Table 4).

**Table 4:** Factors associated with female victimization and male perpetration of IPV (N = 2,689).

Variables			Female Victimization			Male Perpetration		
			OR	95% CI	*p*	OR	95% CI	*p*
Personal history	Witnessing parents’ violence in childhood	Yes	2.92	1.84–4.63	< .001	2.64	1.62–4.32	< .001
		No	1			1		
	Childhood maltreatment by parents	Yes	1.70	1.03–2.82	.040	1.20	0.70–2.05	.508
		No	1			1		
Micro- system	Structure of decision making with partner		1.40	0.85–2.30	.184	0.87	0.54–1.42	.584
	Age (years)	19–39	1.55	0.60–4.02	.371	1.30	0.58–2.90	.527
		40–49	2.09	0.86–5.09	.106	1.67	0.82–3.40	.159
		50–59	1.56	0.69–3.54	.289	1.19	0.62–2.28	.604
		≥60	1			1		
Exo- system	Education level	≤Elementary	0.41	0.14–1.22	.110	1.70	0.57–5.04	.337
		Middle school	0.86	0.35–2.08	.733	1.17	0.43–3.17	.759
		High school	0.77	0.46–1.29	.315	1.64	0.93–2.90	.088
		≥College	1			1		
	Household income (1,000 KRW)	<2,000	0.90	0.30–2.73	.859	1.27	0.58–2.81	.551
		2,000–2,999	1.97	1.10–3.55	.023	1.05	0.55–2.00	.879
		3,000–3,999	0.80	0.48–1.35	.410	1.41	0.85–2.33	.181
		≥4,000	1			1		
	Occupation type	Professional or Manager	0.62	0.24–1.58	.313	1.74	0.81–3.76	.156
		Office job	0.47	0.26–0.84	.011	1.37	0.76–2.46	.301
		Others	1			1		
Macro- system	Attitude to gender roles		1.19	0.78–1.82	.428	0.80	0.53–1.18	.258
	Awareness of neighborhood and community		1.18	0.79–1.74	.416	1.33	0.88–2.02	.178
	Attitude to IPV		2.25	1.40–3.61	.001	2.68	1.59–4.52	< .001
	Awareness of IPV -related laws and policies		0.91	0.80–1.02	.100	0..91	0.82–1.01	.073
	Awareness of support facilities		1.00	0.85–1.17	.992	1.05	0.89–1.24	.537
	F			5.345			2.49	
	*p*			< .001			< .001	
	R^2^			.165			.104	

IPV: Intimate partner violence.

The results of the multiple regression analysis on the men participants showed that 10.4% of the variance was explained by witnessing parents’ violence in childhood and attitude to IPV. The IPV perpetration rate among men who witnessed parental violence in childhood was 2.64 times (OR = 2.64, CI: 1.62–4.32) higher compared with men who did not. In terms of attitudes toward IPV, each point (1–4 points) increase on the mean attitude toward IPV was found to be associated with a 2.68-fold increase in IPV perpetration rate (OR = 2.68, CI: 1.59–4.52) (Table 4).

## Discussion

This study identified the risk factors that affect the female victimization and male perpetration of IPV in Korea to provide data for interventions and policy formation for IPV prevention and response. The prevalence of female victimization in the past 12 months as reported by women in 2016 DVS was 12.1%, showing a decrease compared with 39.1% in 2010 DVS and 29.8% in 2013 DVS. In contrast, the prevalence of male perpetration as reported by men was 11.6% in 2016 DVS [12]. Although male perpetration data reported by men from 2010 and 2013 DVS cannot be found, we hypothesized these to be similar to the prevalence rates reported by women. Thus, the prevalence rates of female victimization of IPV is gradually decreasing in Korea. This decline is interpreted to be a result of the multi-dimensional efforts to prevent partner violence for more than 20 years since the enactment of the Act on the Prevention of Domestic Violence and Protection, etc. of Victims, along with Korea’s economic growth and women’s advancement in society [26]. The growing awareness of gender equality owing to the expansion of new family policies and institutional changes based on the emphasis on democratic and equal gender relations in family and society [27] may have also helped. Meanwhile, the reports of analyzing 2010 DVS and 2016 DVS showed that awareness of IPV- related polices and laws and the willingness to report IPV gradually increased, consistent with the decrease in female victim prevalence [12]. However, the attitude toward patriarchal gender roles still remained the same, particularly among men, showing the need for continued effort [12]. It is important to consider the possibility that the decrease in the prevalence of IPV in South Korea is the result of survey participants being less likely to disclose personal aspects of IPV. Societal stigma related to the victimization and perpetration of IPV has changed with the shift in societal attitudes and female victims’ feelings of shame or embarrassment [28].

Our study found that the occurrence of female victimization and male perpetration were about three times as high in men and women who have witnessed their parents’ violence. This finding is consistent with many previous studies; witnessing parents’ violence in childhood is an important or the strongest risk factor of IPV occurrence in both victimization of women and perpetration by men [5,20,29,30]. In addition, research on the risk factors of men’s lifetime perpetration [16,31] and on IPV victimization of women [32] has also showed that witnessing parents’ violence is an important risk factor of IPV occurrence. The strength and significance of the correlation between witnessing of inter-parental violence and IPV perpetration and victimization suggest the intergenerational transmission of behaviors and gender norms [16,33]. Observation and imitation occur throughout the lifespan but can be particularly important for children and adolescents. Interrupting this cycle is critical to reducing perpetration [16].

Next, we found that women who experienced childhood maltreatment by their parents in childhood were twice as likely to become victims of IPV compared with women who did not. This finding is consistent with many previous studies, which reported that abuse experience in childhood is an important risk factor of IPV occurrence in abused women [1,20,29,30,32]. This finding highlights how childhood experiences influence the likelihood of people later becoming perpetrators or victims of IPV, as well as the need for early childhood interventions, especially for children growing up in families where there is abuse [2]. According to a systematic review of the predictors of DV perpetration and victimization, abuse and family of origin problems experienced in childhood and adolescence, such as witnessing inter-parental abuse and experiencing maltreatment from parents, are consistent predictors of DV perpetration and victimization for men and women [33]. An increasing number of findings show that parental programs, including home visits and education, can reduce child abuse and have an increased effect of reducing children’s violent behavior in the future. Therefore, parental programs to prevent and intervene in partner violence between parents are necessary because such initiatives would address not only parents’ partner violence but also maltreatment of children [34].

On the other hand, our results showed that men’s experience with violence from parents in their childhood had a significant relation with IPV but was not a risk factor that affected IPV occurrence. This was not consistent with previous findings [1,4,20,30]. A study on 242 Korean men showed that men’s experience with violence in their childhood is a risk factor that affects the occurrence of male perpetration [35]. Given the inconsistent results, additional research is necessary.

Attitude toward IPV was analyzed as an important risk factor for women (2.25) and men (2.68) in this study. Our finding was consistent with many previous studies, which reported that beliefs and attitudes about IPV are related to the occurrence of IPV for men and women [1,5,20,29,36,37]. Men who believe wife-beating is acceptable are more than four times as likely to report recent violence against their wives [38,39]. According to men’s lifetime IPV studies in eight low- and middle-income countries, tolerant attitudes toward IPV and inequitable gender attitudes are associated with a higher likelihood of ever perpetrating physical IPV [16]. A study of Korean adults found that men are more tolerant toward IPV compared with women, and perpetrators are likely to victim-blame for the cause of perpetration and have lower awareness of responsibility toward the violence. Further, in the same study, both men and women score low on the item, “Men who perpetrate violence need to take responsibility for their behaviors,” and lack awareness that domestic violence is not right. This implies a possibility of continued female victim by partners in the future [40]. Patriarchal societies, in general, view men as entitled to greater power, privilege, and control of women and children, and consequently, the right to punish them for perceived misbehavior [41]. These results are interpreted to have been found because Korea’s social system and family norms continue [42] to adhere to the long-maintained patriarchal culture, despite the recent change in attitudes toward gender roles.

Overall, when surveying Korean adults regarding their attitudes toward IPV, participants presented a tolerant attitude toward IPV, with gender having the greatest influence on these attitudes. Men have more condoning and tolerant attitudes toward IPV than women. It is common among Korean men to blame female victims rather than to think that violence toward them is the real problem [40]. As women’s tolerant attitude toward IPV is associated with their actual experience of being a victim, changes in the attitude toward partner violence are important [43]. Because the attitude and cultural norm of IPV being acceptable are the most significant factors related to the likelihood of violent behavior [44], efforts to change this tolerant attitude in men and women are necessary.

In Korea, society upholds a mixture of long-held Confucian traditions and modern values. As such, differences in the roles of men and women are attributed to the superior male authority in society and at home. Thus, the gender role norms and violence-tolerant cultures of society affect the formation of individual violence-tolerant attitudes, and in turn, individuals’ learned perception and attitude sustain the norms and cultures of society. In other words, while the perception about partner violence is individualistic, it is socially formed and maintained because the individual learns and embraces the gender role norms and tolerance toward the violence of society [12]. Further, witnessing or experiencing abuse during childhood can lead to a tolerant attitude toward IPV, and thus has an intergenerational transmission effect that leads to spousal abuse. Therefore, this link needs to be managed [4]. In interpreting our analysis in relation to social-ecological theory has led to the observation that female victims of IPV are affected by both their personal history (such as witnessing inter-parental violence or maltreatment by parents in their own childhoods) and the attitudes toward IPV at a macro-system level. Attitudes toward IPV are related to male-centered gender inequality and discrimination against women, which still persist in Korean homes and society. Korean society is both more conservative and patriarchal than that of Western, and even Japanese cultures [45]. As can see from the gender role attitude score observed in this study, women’s traditional values are weakening; however, a significant number of men still exhibit and adhere to a patriarchal attitude [45]—an attitude which is a predictor of violence toward women [45]. Women’s educational background and economic activities, in particular, have increased, but the role of men in the home has not changed considerably, presenting a double burden for women [46]. Although the sense of responsibility to support parents-in-laws at home has weakened, the uneven gender roles in marital relations have hardly changed, while the obligation to educate children is on the rise [47]. These results demonstrate that unequal roles persist between men and women within families, indicating that patriarchal norms persist among nuclear families. In our study, we analyzed “Attitudes toward gender roles” in DVS at the macro-system level and “The structure of decision-making between partners” at the micro-system level. However, these were not related to the occurrence of violence toward women. Therefore, additional research is needed on the socialization process that affects IPV attitudes.

Shedding light on the predictors of IPV occurrence and performing effective interventions should not be separated from the cultural context of the community [48]. Therefore, awareness-enhancing education programs and interventions to prevent IPV need to be provided in various ways that reflect the gender and life cycle differences at the individual and societal levels based on these results.

Finally, many previous studies have shown that low socioeconomic status(SES) was associated with high rate of IPV in both victimization of women and perpetration by men [1,5,20,49]. On the other hand, our study found that low SES affected the rate of female victimization but not male perpetration. This finding is consistent with previous studies, which reported that women from low socioeconomic backgrounds had higher victimization of IPV [1,5,20,49]. In Korea, as of 2014, men’s participation in economic activities was 74%, compared to 51.3% among women. The proportion of non-regular workers among female wage workers was 39.9% (26.5% for men), and the ratio of women’s wage in contrast to that of men’s in similar jobs was only 67%. The rate of career disconnection among married working women accounts for a high rate of 47% [50]. These results suggest that the economically unfavorable situation of women in Korea—whereby women have lower employment rates and incomes than men—may act as a contributing factor to withstand IPV if it occurs. To reduce IPV victimization, policy measures are needed to ensure women’s stable jobs and income such as ensuring equal employment opportunities, salary and minimum wages.

In sum, the common risk factors of IPV in women and men were witnessing inter-parent violence in childhood and tolerance toward IPV. Another risk factor was the experience of maltreatment from parents, but only for women. Korean women remain underprivileged compared with men both in society and in the family. Thus, the witnessing of parents, who have authority, being violent and the experience with maltreatment from parents in childhood link to the tolerance of the abuse of husbands, who have authority, when they become adults. In contrast, men’s experience of maltreatment in childhood does not link to the perpetration of violence as husbands with authority; it is the tolerant attitude toward IPV that has a greater effect on IPV occurrence. In other words, it can be interpreted that both men and women are affected by witnessing their parents’ relationships in their childhood and by the changes in the social attitudes toward IPV. after the enactment of the Act on the Prevention of Domestic Violence and Protection, etc. of Victims. However, compared with men, women also have risk factors of IPV victimization such as experience with maltreatment from parents in their childhood, low socioeconomic status, clearly showing women’s vulnerability.

In this study, personal history and macrosystem level factors had remarkable effects on the occurrence of IPV for both men and women. For women, exosystem level factors also affected IPV occurrence. The number of risk factors is greater for the victimized women compared with the perpetrators men. Thus, it is necessary to strengthen the interventions and policies comprehensively to reduce these factors at multiple levels. Primarily, focus should be on changing harmful social norms through the education and campaign for gender equality and non-violence for both men and women across the life cycle from a young age and on providing various services to parents and children by detecting family violence early [51]. Further, it is necessary to establish integrated policies that consider women’s stable work and income.

## Limitations

This study provides a comparison of the risk factors that affect the occurrence of IPV from the aspects of both female victims and male perpetrators, suggesting a comprehensive yet differentiated direction of intervention on both sides. As this study is an SDA research using DVS, there were limited items in each level. In particular, the microsystem level had only a few items, which could have led to the lack of significant results. Therefore, it is necessary to add the items common in other studies. Further, as this study used a cross-sectional design, it was not possible to derive causal relations. Moreover, the use of national data based on the Act on the Prevention of Domestic Violence and Protection, etc. of Victims limited the sample to only those in legal marital or de facto relationships; thus, the study did not include adults who were divorced or dating. Further, the participants in the DVS are divided into men and women instead of couples. Thus, we could only analyze the results by sex; an integrative analysis of the risk factors that affect the IPV occurrence among women and men could not be undertaken. Owing to the limited SDA research on IPV, general research findings were mostly used in the discussion. In particular, the observations from the IPV prevalence survey indicate that there is a possibility of intentional under-reporting of IPV occurrence due to the fear of retribution and feelings of shame or embarrassment felt by female victims. Although there are cases of over-reported IPV—such as to obtain secondary gains or favorable results from divorce [52], DVS surveyed at the national level is likely to be underreported; this, as such, should be considered when interpreting the reduction in prevalence of cases. Because longitudinal survey data capture is more likely to provide an accurate representation of the population prevalence of IPV [28], it would be beneficial if future cohort survey studies were conducted to identify the accuracy of IPV occurrence.

## Conclusions

There are common and different areas between the risk factors that affect IPV occurrence among countries and between genders. Our findings contribute to the understanding of risk factors for IPV occurrence in Korean men and women. This study revealed that witnessing inter-parent violence in childhood and the attitude toward IPV were strong risk factors of IPV in both men and women. In contrast, the experience of maltreatment from parents in childhood was a risk factor only for women. For women, witnessing inter-parent abuse in childhood, experience with maltreatment from parents in childhood, tolerant attitude toward IPV, low income, and certain occupations were risk factors of IPV. In contrast, for men, only witnessing inter-parent abuse in childhood and tolerant attitude toward IPV were risk factors of IPV. These results support the previous findings that witnessing inter-parent violence and experiencing maltreatment from parents in childhood lead to the internalization of violence, leading to the tolerant attitude toward family violence, and in adult women, to accepting abuse from husbands. In contrast, men showed differences in these risk factors. Additional in-depth research may identify the causes for these differences. Further, because the trend of prevalence of female victimization in Korea is decreasing, it is necessary to explore the future direction of interventions and support through research that examines the specific contributors to the changes in these trends. It is also necessary to seek a differentiated approach from the perpetrator’s perspective through the accumulation of DVS research data on men perpetrators.

In the future, IPV prevention programs should increase focus on transforming gender norms and attitudes toward IPV and addressing childhood abuse. To change the social attitude toward IPV, education and campaigns should be strengthened throughout the life cycle from a young age for both men and women. IPV-related service providers and policy makers may need to consider childhood family violence history in both men and women in the context of IPV. Moreover, the importance of strategies and policies to empower women socio-economically and personally needs to be recognized. In particular, as women victims are affected by more risk factors than men perpetrators, it is necessary to establish comprehensive policies that consider the risk factors at the ecological levels.
